# Epigallocatechin-3-gallate protects porcine oocytes against post-ovulatory aging through inhibition of oxidative stress

**DOI:** 10.18632/aging.204368

**Published:** 2022-11-14

**Authors:** Dongjie Zhou, Ming-Hong Sun, Wen-Jie Jiang, Xiao-Han Li, Song-Hee Lee, Geun Heo, Ying-Jie Niu, Sun A. Ock, Xiang-Shun Cui

**Affiliations:** 1 Department of Animal Science, Chungbuk National University, Cheongju, South Korea; 2 Joint International Research Laboratory of Agriculture and Agri-Product Safety of the Ministry of Education of China, Yangzhou University, Yangzhou, China; 3 Animal Biotechnology Division, National Institute of Animal Science, Rural Development Administration, Jeonju, South Korea

**Keywords:** epigallocatechin-3-gallate, mitochondria, oocyte, oxidative stress, post-ovulatory aging

## Abstract

Increased levels of oxidative stress are major factors that drive the process of post-ovulatory oocyte aging. Epigallocatechin-3-gallate (EGCG), which accounts for up to 50% of the catechins, possesses versatile biological functions, including preventing or treating diabetes, cancer, and heart diseases. The aim of this study was to explore whether EGCG can delay porcine oocyte aging by preventing oxidative stress. Metaphase II (MII) oocytes were cultured for 48 h with different concentrations of EGCG (0–100 μM) *in vitro* as a post-ovulatory aging model. An optimal concentration of 5 μM EGCG maintained oocyte morphology and developmental competence during aging. The oocytes were randomly divided into five groups: fresh, 24 h control, 24 h EGCG, 48 h control, and 48 h EGCG. The results suggest that EGCG significantly prevents aging-induced oxidative stress, glutathione (GSH) reduction, apoptosis, and autophagy. Moreover, mitochondria DNA copy number was decreased, and the number of active mitochondria and adenosine triphosphate (ATP) levels significantly increased by supplementation with EGCG. Thus, EGCG has a preventive role against aging in porcine post-ovulatory oocytes due to its ability to inhibit oxidative stress and promote mitochondrial biogenesis.

## INTRODUCTION

The “post-ovulatory aging” refers to the oocytes released from the ovary. Over time, the cytoplasm continues to age and eventually die, which is inevitable [1]. Post-ovulatory aging causes many defects, such as cortical granules partial exocytosis [2, 3], zona pellucida hardening [3, 4], a decline in MPF and MAPK levels [5], cytoskeleton abnormalities, and chromosome condensation [6]. Post-ovulatory aging induces oxidative stress which causes mitochondrial dysfunction, apoptosis due to the activation of caspases [7–9], calcium ions homeostasis perturbation, oxidative damage to lipids, proteins, and DNA components of the cell [10], which finally induce epigenetic changes [11]. Moreover, post-ovulatory aging associates with decreased the fertilization rate, poor embryo quality and increased abnormalities in offspring. In additional, uncovering the mechanisms of post-ovulatory oocyte aging to develop strategies to prevent or delay post-ovulatory oocyte aging and increase the time required to manipulate oocytes for *in vitro* fertilization (IVF) and human assisted reproductive technologies [12].

Epigallocatechin-3-gallate (EGCG), a major polyphenol in green tea, is responsible for the several health benefits such as antioxidation, induction of apoptosis, and inhibition of angiogenesis and metastasis [13]. EGCG mediates its effects by altering cell membrane and intracellular protein, cell signaling molecule, and microRNA profiles. In addition, previous studies have demonstrated that rats treated with EGCG exhibit a significantly longer lifetime, accompanied by a reduction in oxidative stress and inflammation on account of activation of the proteins involved in the regulation of longevity, including forkhead box O3 (FOXO3a) and Sirtuin 1 (SIRT1) [14]. Furthermore, studies from both invertebrate and mammalian model organisms have suggested an increase in the lifespan of an organism upon EGCG treatment [15–17]. The study about the effect of natural compounds like EGCG on the post-ovulatory aging process in oocytes has not been reported. In the present study, we have evaluated the role and underlying mechanism of EGCG in delaying the post-ovulatory oocyte aging in pigs. After selecting oocytes with first polar body, the MII stage oocytes were cultured for aging with or without EGCG for 24 h or 48 h. The supplement of 5–10 μM EGCG could prevent oocyte fragmentation and maintain the ability of embryo pre-implantation development, and prevent mitochondrial dysfunction, apoptosis, and autophagy induced by post-ovulatory oocyte aging.

## MATERIALS AND METHODS

All animal work was conducted according to the Institutional Animal Care and Use Committee guidelines under currently approved protocols at Chungbuk National University. All chemicals sodium pyruvate, epidermal growth factor (EGF), luteinizing hormone (LH), follicle-stimulating hormone (FSH), calcium chloride (CaCl_2_), magnesium sulfate (MgSO_4_), polyvinyl alcohol (PVA) and paraformaldehyde (PFA) were purchased from Sigma-Aldrich Corporation, Inc. (St. Louis, MO, USA) unless otherwise indicated. All manipulations were performed on a heat plate at 38.5°C.

### Collection of porcine cumulus–oocyte complexes (COCs) and *in vitro* maturation (IVM)

Ovaries from pigs were collected from a local slaughterhouse (Farm Story Dodarm B&F, Umsung, Chungbuk, Korea) and transported to the laboratory in pre-warmed NaCl solution with 75 mg/mL penicillin G and 50 mg/mL streptomycin sulfate. The porcine follicles with around 3–6 mm diameter were aspirated by using a 10-mL disposable syringe. COCs with more than two layers of compact cumulus cells (CCs) were selected and washed three times with an IVM medium [TCM-199 (11150–059; Gibco, Grand Island, NY, USA) supplemented with 100 mg/L sodium pyruvate, 10 ng/mL EGF, 10% (v/v) porcine follicular fluid, 10 IU/mL LH, and 10 IU/mL FSH]. Finally, 50–100 COCs per well were cultured in 4-well dishes covered with mineral oil for 44–48 h until maturation to the MII phase at 38.5°C with 5% CO_2_ [18].

### *In vitro* aging (IVA) and supplementation with EGCG

The CCs were removed in 1 mg/mL hyaluronidase by pipetting for approximately 40 times. The MII stage oocytes were selected with first polar bodies for further studies. For analysis of oocyte post-ovulatory aging, the selected MII stage oocytes were continuous cultured in IVM medium with or without EGCG covered with mineral oil for an additional 24 or 48 h. The oocyte fragmentation rate was calculated at 24 and 48 h after IVA.

### Parthenogenetic activation and *in vitro* culture (IVC)

According to previous study [19], two direct-current pulses (PDC) of 110 V for 60 μs were used for the parthenogenetic activation of the fresh and aged MII oocytes in 297 mM mannitol (pH 7.2) containing 0.1 mM CaCl_2_, 0.05 mM MgSO_4_, 0.01% PVA (w/v), and 0.5 mM HEPES. The activated oocytes were treated with 7.5 μg/mL cytochalasin B in bicarbonate-buffered porcine zygote medium 5 (PZM-5) containing 4 mg/mL BSA for 3 h to inhibit the pseudo-second polar body extrusion. The oocytes were then thoroughly washed and cultured in 4-well plates with bicarbonate-buffered PZM-5 containing 4 mg/mL BSA for 6 d at 38.5°C (5% CO_2_). The blastocyst rate was analyzed on day 7, and the quality of the blastocysts was evaluated as described by Gardner [20].

### Glutathione (GSH) and reactive oxygen species (ROS) measurements

For GSH level detection, ten oocytes from each group were stained with 10 μM 4-chloromethyl-6,8-difluoro-7-hydroxycoumarin dye (CellTracker™ Blue CMF2HC; Thermo Fisher Scientific, Waltham, USA) at 38.5°C for 30 min and then washed three times with PBS/PVA. For ROS level determination, ten oocytes from each group were incubated with 10 μM 2,7-dichlorodihydrofluorescein diacetate (H2DCF-DA, Cat #D399; Molecular Probes, Eugene, OR, USA) at 38.5°C for 30 min. The fluorescence was detected using a digital camera (DP72; Olympus, Tokyo, Japan) connected to a fluorescence microscope (IX70; Olympus). The fluorescence intensity of oocytes was analyzed using ImageJ software version 1.44 g (National Institutes of Health, Bethesda, MD, USA) to quantify GSH and ROS levels [21].

### Immunofluorescence and confocal microscopy

As previously reported [22], oocytes were fixed with 3.7% PFA for 30 min at room temperature (20–25°C) after washing with PBS/PVA solution thrice, and then permeabilized with 1% Triton X-100 for 30 min and blocked in 3.0% BSA containing 0.1% Triton X-100 for 1 h at room temperature. These oocytes were incubated at 4°C overnight with anti-TOM20 (1:50, F-10, Cat # SC-17764; Santa Cruz Biotechnology, Santa Cruz, CA, USA), anti-Beclin 1 (1:50; 11306-1-AP; ProteinTech, Wu Han, China), anti-active-caspase 3 (1:50, C8487; Sigma), or anti-p53 (1:50; SC6243; Santa Cruz Biotechnology) diluted with 3.0% BSA with 0.1% Triton X-100. After washing with PBS/PVA thrice, the oocytes were incubated with Alexa Fluor 488™ donkey anti-mouse immunoglobulin G (IgG) (H+L) (1:200; Cat # A21202; Invitrogen, Carlsbad, CA, USA) or Alexa Fluor 546™ donkey anti-rabbit IgG (H+L) (1:200; Cat # A10040, Invitrogen) for 1 h at room temperature. The oocytes were mounted onto slides using Vectashield mounting medium with DAPI (Vector Laboratories, Burlingame, CA, USA) and examined under a confocal microscope (Zeiss LSM 710 META, Jena, Germany). Images were processed by Zen software (version 8.0, Zeiss).

### Western blot analysis

As previously report [23], a total of approximately 100 porcine oocytes per group were lysis with 10 μl RIPA buffer and 10 μl loading buffer, and then heated at 98 C for 10 min. Lysates were separated by 6–12% SDS-PAGE gel and transferred onto polyvinylidene fluoride membranes. Next, the membranes were blocked with 5% skim milk in TBST buffer for 1 h and then incubated with anti-PINK1, DRP1, LC3 or -GAPDH antibody at 4°C overnight. Subsequently, the membranes were washed with TBST buffer thrice and incubated at room temperature for 1 h with horseradish peroxidase-conjugated goat anti-mouse IgG or goat anti-rabbit IgG (1:20,000; Santa Cruz Biotechnology). Blots were visualized with SuperSignal™ West Femto Maximum Sensitivity Substrate (Thermo Fisher Scientific, Waltham, USA) using a charge-coupled device camera and UviSoft software (Uvitec, Cambridge, United Kingdom).

### Active mitochondrial staining

Oocytes were stained in 500 nM MitoTracker Red CMXRos (Cat #M7512; Invitrogen) for 30 min at 38.5°C. After washing with PBS/PVA thrice, TOM20 was stained as described in the immunofluorescence and confocal microscopy subsection.

### Mitochondrial DNA (mtDNA) copy number measurements

Three oocytes were sampled into a PCR tube with 8 μl of lysis buffer (20 mM Tris, 0.4 mg/mL proteinase K, 0.9% Nonidet-40, and 0.9% Tween 20) and incubated at 65°C for 30 min, 95°C for 5 min. After dilution with H_2_O for 1:25, real-time qPCR was performed with WizPure qPCR Master (W1731-8; Wizbio Solutions, Seongnam, South Korea) according to the manufacturer’s instructions, on a QuantStudio™ 6 Flex Real-Time PCR System (Applied Biosystems, Waltham, MA, USA). The target gene ND1 which primers were ND1-F (5′-CCT ACT GGC CGT AGC ATT CC-3′), ND1-R (5′-GAG GAT GTG CCT GGT CGT AG-3′) was amplified as follows: 95°C for 3 min, followed by 40 cycles of 95°C for 15 s, 60°C for 25 s, 72°C for 15 s, and a final extension at 72°C for 5 min. The mRNA quantification data were analyzed using the 2^−ΔΔCt^ method. CT values were relative to the fresh group.

### Adenosine triphosphate (ATP) measurements

ATP level was detected by the luciferin–luciferase ATP assay system with a luminometer (CentroPRO LB 962; Berthold, ND, USA) according to the ATP determination kit (A22066; Molecular Probes) manufacturer’s instructions. Briefly, ten oocytes per group were sampled in a PCR tube containing 30 μl of lysis buffer (20 mM Tris, 0.9% Nonidet-40, and 0.9% Tween 20). The oocytes were homogenized by vortexing until lysis occurred. The standard reaction solution was prepared following the manufacturer’s instructions and kept on ice and avoided the light before detection. 5 μl of the lysates were added to 96-well plates and equilibrated for 10 s. Subsequently, 150 μl of the standard reaction solution was mixed with each sample, and the optical signal was integrated for 10 s after a delay of 2 s. The light intensity in the fresh group was arbitrarily assigned a value of 1, and the relative light intensity in the aging group was then measured.

### Statistical analysis

All of experiments were repeated at least three times, and immunofluorescence representative images are shown in the figures. In statistics, one-way analysis of variance (ANOVA) or Student’s *t*-test was used for data analysis. All percentage data were presented as the mean ± standard error of the mean (SEM). Statistical significance was set at *p* < 0.05.

### Data availability statement

The original contributions presented in the study are included in the article, further inquiries can be directed to the corresponding author.

## RESULTS

### EGCG prevents fragmentation of porcine oocytes induced by aging

**Figure 1:**
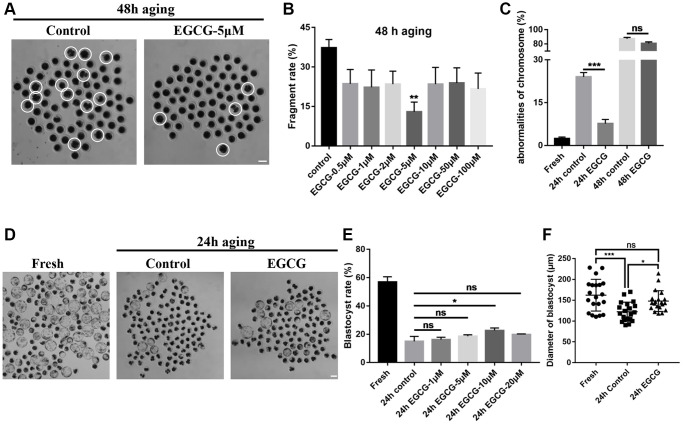
**Epigallocatechin-3-gallate (EGCG) rescues aging-induced fragmentation of porcine oocytes.** (**A**) Oocyte morphology after 48 h of aging with/without EGCG. The white dotted circle indicates fragmented oocytes. Scale bars represent 100 μm. (**B**) Fragmentation rate upon treatment with different concentrations of EGCG following aging for 48 h. ^**^p < 0.01 indicates significant differences. (**C**) Quantitative analysis revealed chromosome misalignment after aging for 24 h and 48 h. (**D**) The D7 embryo morphologies in the fresh, 24 h control, and 24 h EGCG groups. (**E**) The blastocyst rate upon treatment with different concentrations of EGCG after aging for 24 h. Scale bars represent 100 μm. (**F**) Diameters of blastocyst in the fresh, 24 h control, and 24 h EGCG groups. Values are expressed as the mean ± SEM.

After an IVM step for 44 h, the CCs were removed by pipetting, and MII stage oocytes were selected as fresh oocytes. As a post-ovulatory aging model, MII stage oocytes were continuous cultured in IVM for 24 h or 48 h. The morphology of the oocytes was investigated at 0, 24, and 48 h after IVA (Figure 1A). We found that oocyte fragmentation rate (37.27 ± 3.18%) was much higher at 48 h after IVA (Figure 1B). The ability of EGCG to maintain oocyte morphology during post-ovulatory aging was investigated by treating the oocytes with EGCG at different concentrations (0, 0.5, 1, 2, 5, 10, 50, and 100 μM). The fragmentation rate of the aging oocytes was significantly reduced in the 5 μM EGCG treatment group (15.3 ± 4.80%), compared with those in the 48-h aging control group (37.27 ± 3.18%) (*p* < 0.01, Figure 1B). Both pronuclear like nuclear and highly condensed nuclear were observed as abnormal chromosome after aging. However, the ratio of oocytes with those abnormal chromosomes were lower in EGCG treatment after 24 h aging (Figure 1C). Moreover, the blastocyst formation was partially rescued by supplementation with 10 μM EGCG (22.64 ± 1.05%) compared with the 24 h aging control group (14.96 ± 2.05%) (*p* < 0.01, Figure 1D and 1E). The diameter of blastocysts were significantly reduced in 24 h aged blastocysts compared with those in the fresh group, which was rescued by supplementation with EGCG (122.40 ± 5.147 vs. 162.20 ± 8.52 μm, *P* < 0.005; 122.40 ± 5.147 vs. 148.00 ± 5.57 μm, *P* < 0.05, Figure 1F). Therefore, 5 μM EGCG was selected for further studies.

### EGCG rescues aging-induced oxidative stress

**Figure 2:**
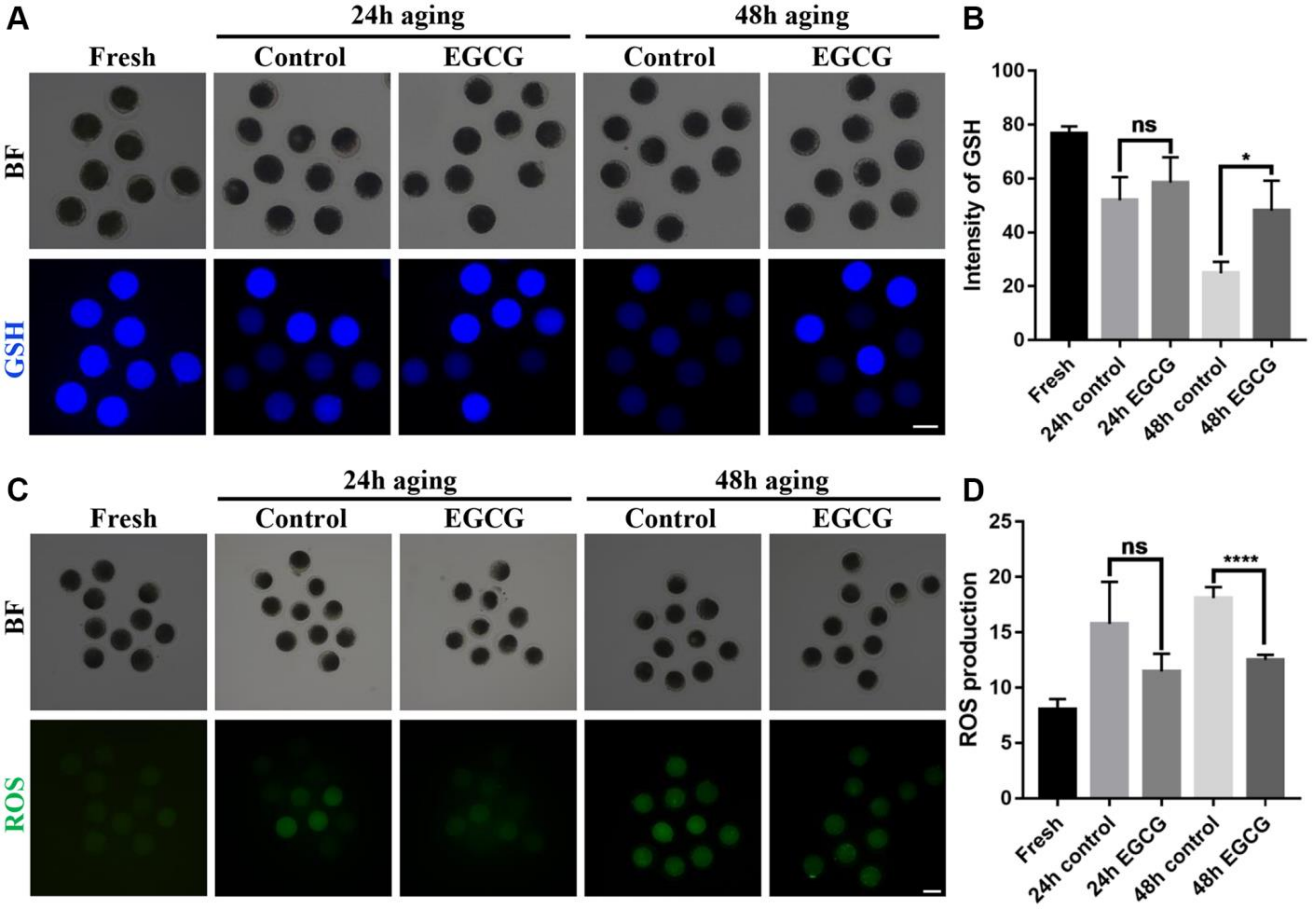
**Epigallocatechin-3-gallate (EGCG) rescues aging-induced oxidative stress.** (**A**) Representative images of glutathione (GSH) in the Fresh, 24 h control, 24 h EGCG, 48 h control, and 48 h EGCG oocytes. (**B**) Quantified intracellular levels of GSH by relative fluorescence intensity (RFI). ^*^p < 0.05 indicates significant difference. (**C**) Representative images of reactive oxygen species (ROS) in the Fresh, 24 h control, 24 h EGCG, 48 h control, and 48 h EGCG oocytes. (**D**) Quantified intracellular levels of ROS by RFI. Scale bars represent 100 μm. Values are presented as the mean ± standard error of the mean (SEM). ^****^p < 0.001 indicates significant difference. Values are expressed as the mean ± SEM.

The antioxidant protective effect of EGCG on aging oocytes was determined *in vitro* by detecting the GSH and ROS levels. As shown in Figure 2A and 2B, the GSH levels were gradually decreased during IVA (*p* < 0.001). However, GSH levels were higher in the 48 h EGCG group compared with those in the 48 h control group (*p* < 0.05). ROS production substantially increased during oocyte IVA (*p* < 0.001). However, ROS levels in the 48 h EGCG group were significantly lower than those in the 48 h control group (*p* < 0.001, Figure 2C and 2D). These data indicate that EGCG can prevent GSH reduction and ROS production induced by oocyte IVA.

### EGCG rescues mitochondrial dysfunction induced by aging

**Figure 3:**
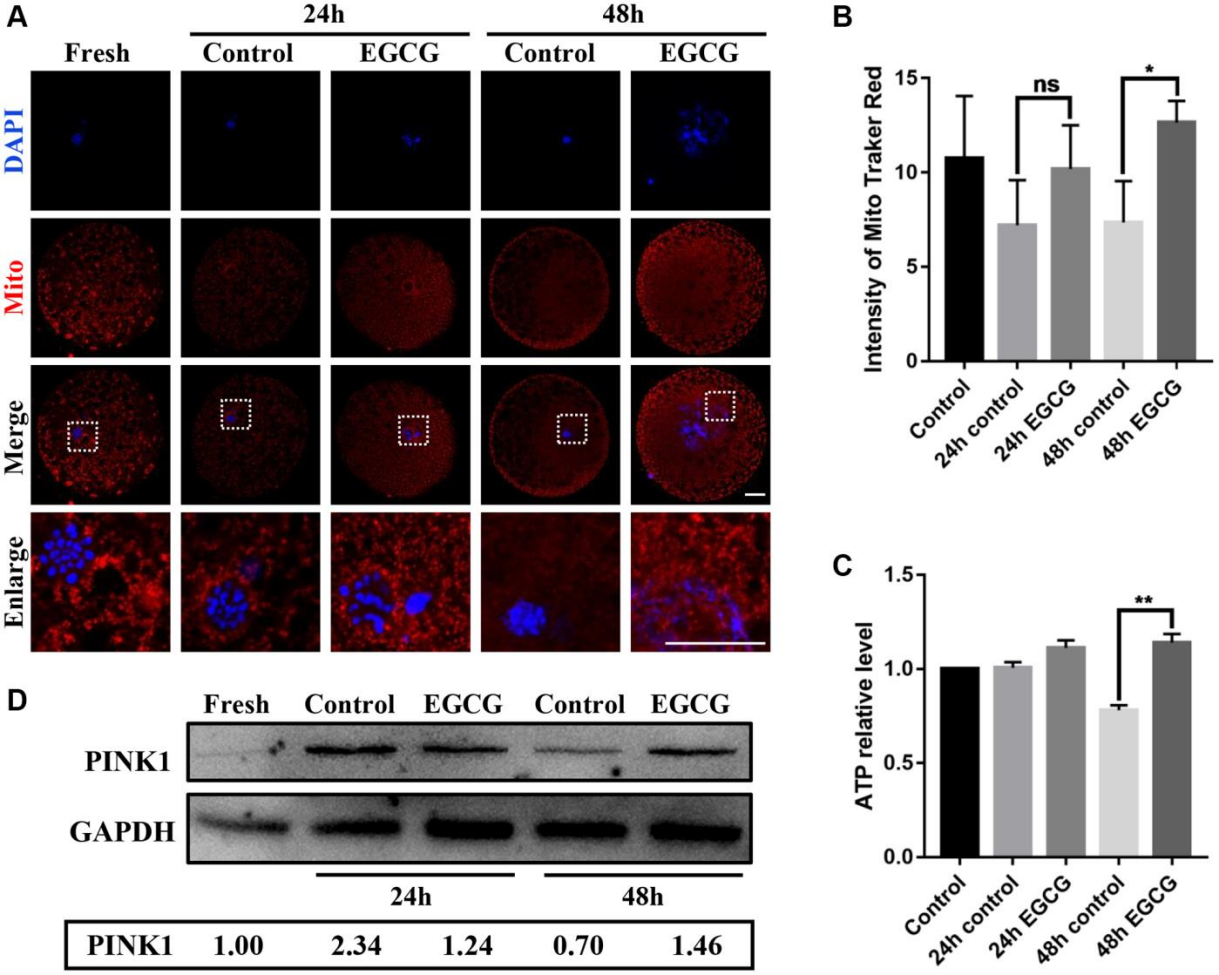
**Epigallocatechin-3-gallate (EGCG) rescues aging-induced mitochondrial dysfunction.** (**A** and **B**) Fluorescence intensity of MitoTracker Red in aging Fresh, 24 h control, 24 h EGCG, 48 h control, and 48 h EGCG oocytes. Scale bars represent 20 μM. (**C**) Adenosine triphosphate (ATP) levels of oocytes in the Fresh, 24 h control, 24 h EGCG, 48 h control, and 48 h EGCG groups. (**D**) Lysates from oocytes with/ without EGCG for 24 h and 48 h aging were analyzed by western blot for detection of the PINK1 and GAPDH. ^*^p < 0.05 and ^**^p < 0.01 indicate significant differences. Values are expressed as the mean ± SEM.

To detect mitochondrial activity, the oocytes were stained with MitoTracker Red CMXRos. As shown in Figure 3A and 3B, mitochondria activity was significantly reduced in oocytes upon IVA by approximately 68% of normal levels (*p* < 0.05). ATP levels were also measured to estimate mitochondrial function. Although the ATP levels did not decrease initially after 24 h IVA (*p* > 0.05), the significant reduction of ATP level was detected after 48 h IVA (*p* < 0.01, Figure 3C). Next, to evaluate if EGCG could rescue mitochondria dysfunction during the post-ovulatory aging of oocytes, the oocytes were treated with 5 μM EGCG for 24 h or 48 h, and the mitochondrial activity and ATP levels were measured. Our results indicated that EGCG significantly prevented the reduction in both mitochondrial activity (*p* < 0.05, Figure 3B) and ATP levels after 48 h IVA. (*p* < 0.01, Figure 3C). Moreover, the level of PINK1 was performed by western blot which was higher in EGCG supplementation group compare with control after 48 h aging (Figure 3D). These data suggest that EGCG has protective effects on the mitochondria function of oocytes during IVA.

### EGCG prevents the release of cytochrome c induced by aging

**Figure 4:**
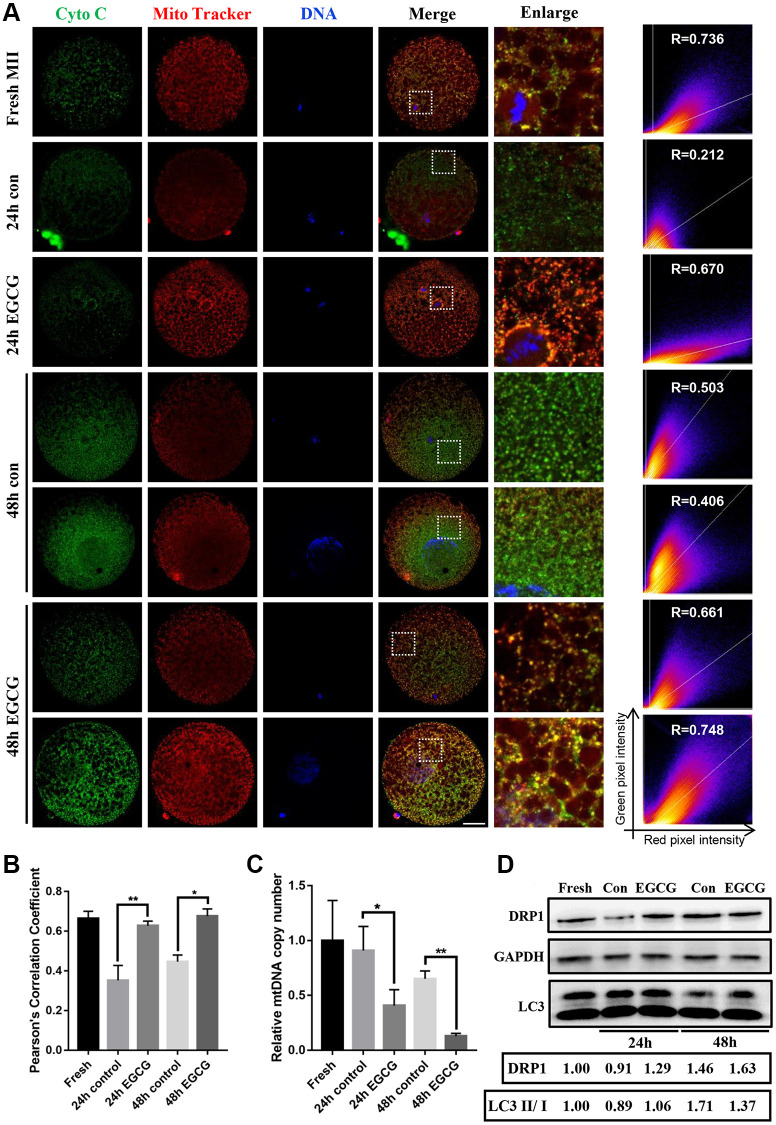
**Epigallocatechin-3-gallate (EGCG) prevents cytochrome *c* release induced by aging.** (**A** and **B**) Immunofluorescence images and Pearson’s correlation coefficient (r) show colocalization of cytochrome *c* and MitoTracker Red in the Fresh, 24 h control, 24 h EGCG, 48 h control, and 48 h EGCG oocytes. Scale bars represent 20 μm. (**C**) Relative mitochondrial DNA (mtDNA) copy number in the Fresh, 24 h control, 24 h EGCG, 48 h control, and 48 h EGCG oocytes. (**D**) Lysates from oocytes with/ without EGCG for 24 h and 48 h aging were analyzed by western blot for detection of the DRP1, LC3 and GAPDH. ^*^p < 0.05 and ^**^p < 0.01 indicate significant difference. Values are expressed as the mean ± SEM.

The role of EGCG treatment on cellular apoptosis was determined by analyzing the colocalization of cytochrome c and mitochondria (Figure 4A and 4B). We found that the disruption of the colocalization of mitochondria and cytochrome c upon IVA in the 24 h and 48 h groups was significantly prevented by supplementation with EGCG. Pearson’s correlation value indicated that supplementation with EGCG resulted in evident colocalization of mitochondria and cytochrome c to prevent apoptosis.

Next, the mtDNA copy number was analyzed using RT-PCR. In agreement with previous study [24], the mtDNA copy number was significantly decreased after 48 h IVA (*p* < 0.001, Figure 4C). However, in EGCG-treated oocytes, the mtDNA copy number was significantly decreased at both 24 and 48 h of IVA but did not increase as predicted compared with control (24 h, *p* < 0.05; 48 h, *p* < 0.01, Figure 4C). Moreover, DRP1 level was higher in EGCG group compared to control group, which could induce mitochondrial fission. LC3 II/I ratio was lower in 48 h EGCG group compared to 48 h control group, which indicated that mitophagy was inhibited by EGCG supplementation. Collectively, the results showed that EGCG prevents cytochrome *c* release and improves mitophagy.

### EGCG rescues aging-induced apoptosis and autophagy

**Figure 5:**
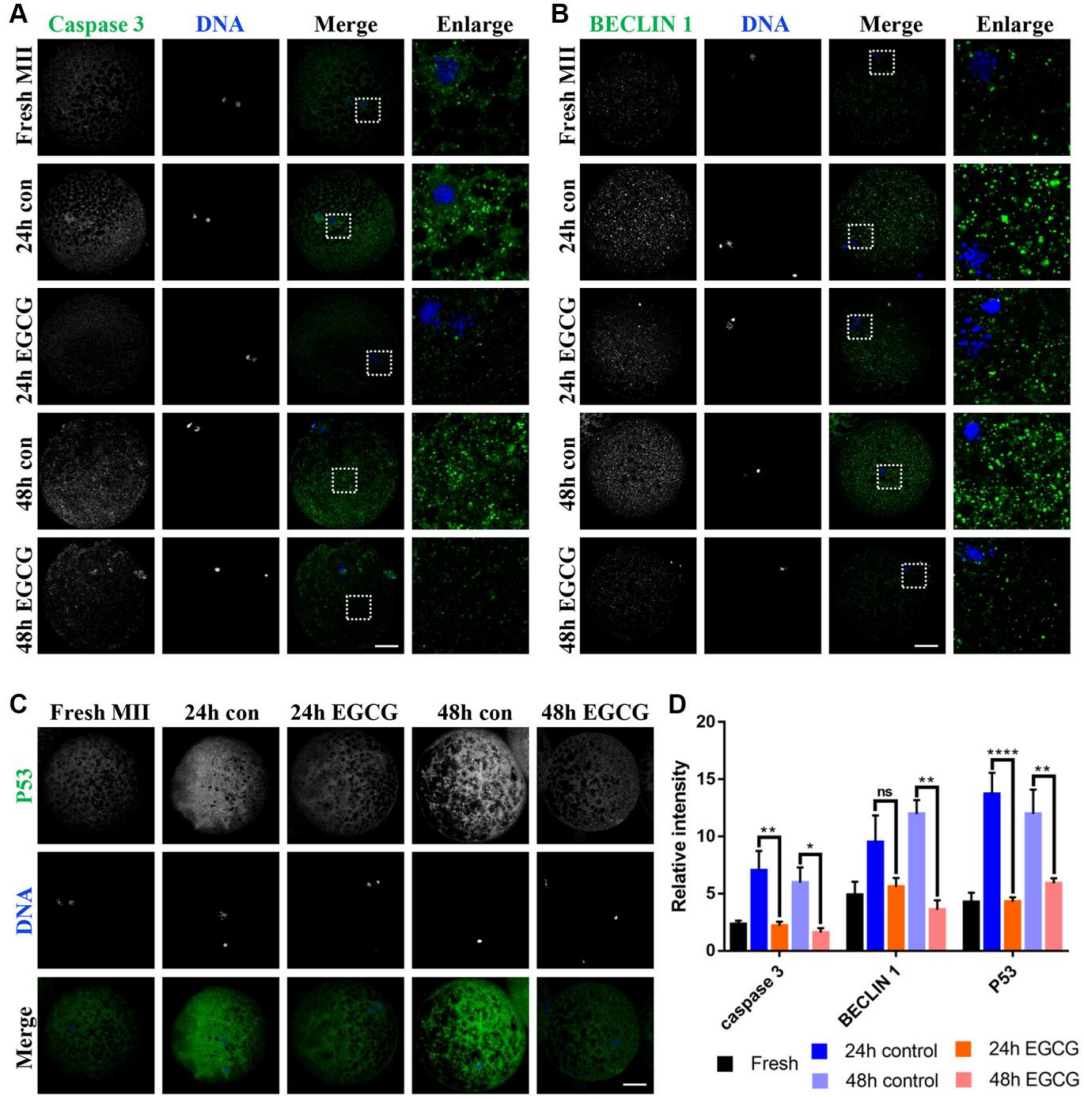
**Epigallocatechin-3-gallate (EGCG) rescues apoptosis and autophagy induced by oocyte aging.** (**A**) Active-caspase 3 expression was analyzed by immunofluorescence in the Fresh, 24 h control, 24 h EGCG, 48 h control, and 48 h EGCG oocytes. (**B**) Beclin 1 level was analyzed by immunofluorescence in the Fresh, 24 h control, 24 h EGCG, 48 h control, and 48 h EGCG oocytes. (**C**) P53 expression was analyzed by immunofluorescence in the Fresh, 24 h control, 24 h EGCG, 48 h control, and 48 h EGCG oocytes. (**D**) Relative intensities of caspase 3, Beclin 1, and P53 in the Fresh, 24 h control, 24 h EGCG, 48 h control, and 48 h EGCG oocytes. Scale bars represent 20 μm. ^*^p < 0.05, ^**^p < 0.01 indicate significant differences between treatment groups. Values are expressed as the mean ± SEM.

Due to the activation of caspases, apoptosis culminated in the process of post-ovulatory aging [1]. Therefore, to evaluate whether apoptosis was prevented by treatment with EGCG, the expression level of P53 and active-caspase 3 was detected by using immunofluorescence. As shown in Figure 5A and 5D, the intensity of active-caspase 3 was higher in aged oocytes than that in fresh oocytes. However, supplementation with EGCG reduced the expression of active-caspase 3 at 24 h (*p* < 0.01) and 48 h (*p* < 0.05) after IVA. Meanwhile, the level of p53 was also decreased after supplementation with EGCG for 24 h (*p* < 0.001) and 48 h of aging (*p* < 0.01, Figure 5C and 5D). Moreover, autophagy is reported to be involved in oocyte aging. We examined whether autophagy was inhibited in aged oocytes. As shown in Figure 5B and 5D, the aged group presented a significantly higher expression of Beclin 1 compared to that in the fresh group, and the level was significantly reduced after treatment with EGCG for 48 h (*p* < 0.01). These results demonstrate that EGCG can prevent post-ovulatory aging-induced apoptosis and is a potent molecule capable of delaying post-ovulatory aging.

## DISCUSSION

**Figure 6:**
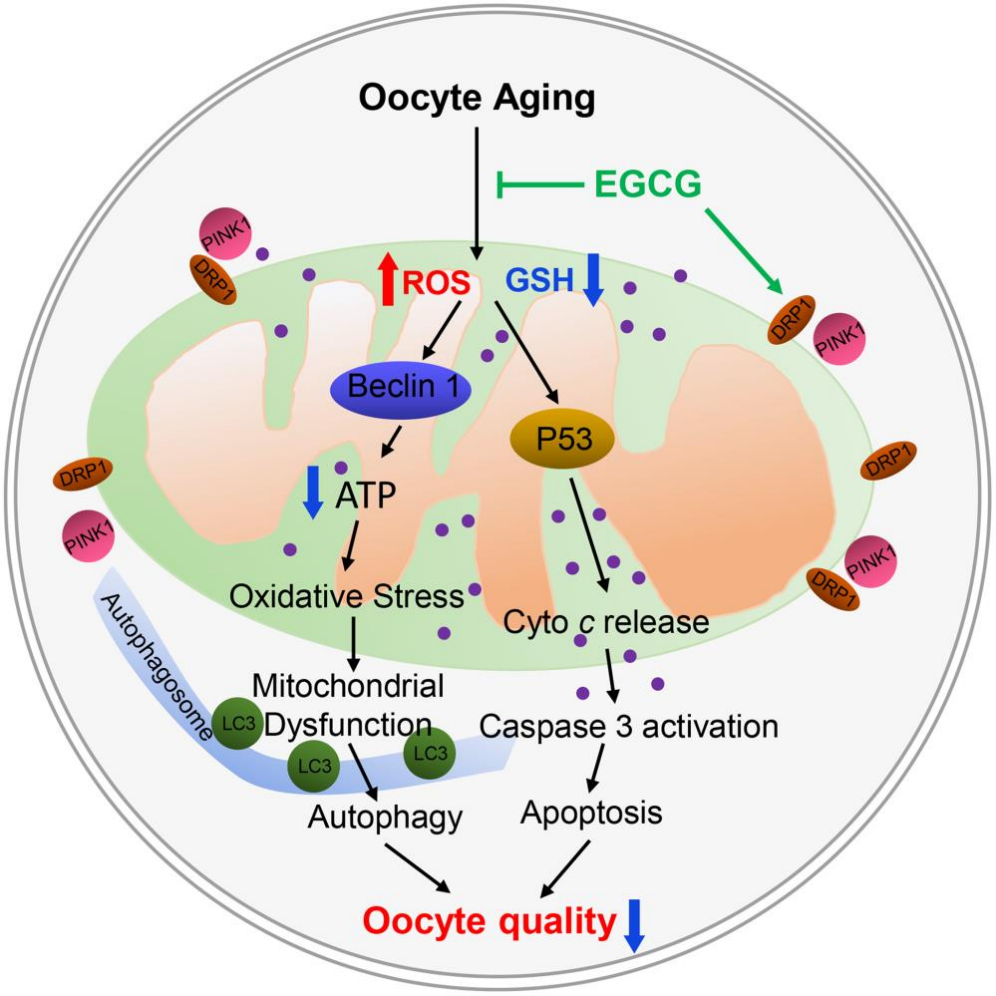
**Schematic representation of the protective effects of epigallocatechin-3-gallate (EGCG) by targeting mitochondrial function after post-ovulatory aging in pig.** EGCG prevents apoptosis by inhibiting mitochondrial P53 and caspase 3 activity and mitochondrial dysfunction and ultimately prevents the oocyte damage induced by post-ovulatory aging.

It is well known that oxidative stress triggers the process of post-ovulatory oocyte aging. In fact, previous studies indicate that antioxidant supplements can delay the post-ovulatory aging process in oocytes [1, 25–30]. In this case, glutathione is an endogenous antioxidant, which prevents the harmful effects of oxidative stress by reducing the accumulation of reactive oxygen species in oocytes. However, when oocytes missed the optimal fertilization time, ovulated MII oocytes showed increased GSH consumption and ROS accumulation [31, 32]. In the present study, when the IVM medium was supplemented with EGCG, ROS production and GSH consumption was abated during the process of post-ovulatory aging (Figure 6). These data suggest that EGCG supplementation can protect oocytes from oxidative stress induced by IVA.

Moreover, oxidative stress in post-ovulatory aged oocytes can induce apoptosis and autophagy [1]. In this study, the supplementation of EGCG prevented autophagy by inhibiting the level of BECLIN1 and LC3, prevented apoptosis by reducing P53 and caspase-3 activation (Figure 6). These results demonstrate that EGCG can inhibit ROS accumulation and prevent the harmful effects of oxidative stress on post-ovulatory aging oocytes. Since EGCG can improve the developmental ability of aging oocytes, it is suggested that EGCG can delay the porcine oocyte post-ovulatory aging.

Previously study showed that mitochondrial activity and ATP production decrease in aged oocytes [33]. EGCG supplementation can, however, rescue these effects by increasing the level of ATP. The EGCG antioxidant properties may be benefit to the mitochondria. However, we found that the mtDNA copy number was reduced after supplementation of EGCG, which may be caused by mitophagy.

Normally, PINK1 translocate to the mitochondria inner membrane and is degraded by the proteasome system [34]. However, PINK1 accumulates in the mitochondrial outer membrane [35] and recruits PARKIN to damaged mitochondrial outer membranes in depolarized mitochondria [36]. PINK1 also interacts with DRP1 to promote mitochondrial fission which regulates the mitochondrial number, size, and morphology with mitochondrial fusion in a dynamic manner [37, 38]. Therefore, EGCG induces PINK1 and DRP1 expression during IVA (Figure 3D and 4D) and accelerating the clearance of damaged mitochondria. Even the number of mitochondria in the EGCG-treated oocytes was decreased; the other mitochondria were healthy. Therefore, healthy mitochondria would further lead to a decrease in the production of ROS.

In addition, autophagy was shown to be downregulated during the EGCG-induced mitophagy process (Figure 4D and 5B). This may be because autophagy is increased due to the elimination of damaged cell components formed by oxidative stress during the autophagy process after aging. Hence, the autophagy was downregulated as an adaptive response. The current study reveals how the mitochondrial quality control (QC) system of post-ovulatory aging oocytes is disrupted, and EGCG supplementation is shown to prevent these processes.

## CONCLUSION

EGCG prevented several cellular alterations induced by post-ovulatory aging and promoted the development ability of aged embryos in pigs. EGCG has the potential to delay the aging of human oocytes or oocytes from other mammalian species processed for clinical assisted reproductive technology.
